# Regulatory Effect of Fucoidan Hydrolysates on Lipopolysaccharide-Induced Inflammation and Intestinal Barrier Dysfunction in Caco-2 and RAW264.7 Cells Co-Cultures

**DOI:** 10.3390/foods13223532

**Published:** 2024-11-05

**Authors:** Xiaodan Fu, Xinru Huang, Huizi Tan, Xiaojun Huang, Shaoping Nie

**Affiliations:** 1 State Key Laboratory of Food Science and Resources, Nanchang University, Nanchang 330047, China; luna_9303@163.com (X.F.); ncuspyhuangxinru@163.com (X.H.); huizi.tan@ncu.edu.cn (H.T.); huangxiaojun0617@163.com (X.H.); 2 China-Canada Joint Laboratory of Food Science and Technology (Nanchang), Nanchang 330047, China; 3 Key Laboratory of Bioactive Polysaccharides of Jiangxi Province, Nanchang 330047, China

**Keywords:** low-molecular-weight hydrolyzed fucoidan, Caco-2/RAW264.7 cell co-culture, inflammation, transcriptome, intestinal barrier

## Abstract

Fucoidan, a sulfated polysaccharide rich in fucose, is derived from brown algae and marine invertebrates. Multiple bioactivities have been shown with fucoidan, while growing attraction has emerged in its low-molecular-weight (Mw) hydrolysates. Here, the anti-inflammatory effect of fucoidan, low-Mw acidolyzed fucoidan (LMAF, <1.5 kDa), and high-Mw acidolyzed fucoidan (HMAF, 1.5–20 kDa) were investigated in vitro using lipopolysaccharide (LPS)-stimulated Caco-2 and RAW264.7 co-cultures. Fucoidan, LMAF, and HMAF with different structures exhibited varied anti-inflammatory effects. LMAF and HMAF effectively decreased the nitric oxide release of RAW264.7 cells. LMAF exhibited a competitive effect in reducing tumor necrosis factor-alpha, interleukin-1 beta, and interleukin-6 levels compared to HMAF and fucoidan. Transcriptome of RAW264.7 revealed that LPS and LMAF mainly regulated the transcriptional expression of genes, including *Tnf*, *Il6*, *Il1b*, *Junb*, and *Nfkb1* in the TNF signaling pathway, NF-kappa B signaling pathway, and cytokine–cytokine receptor interaction. RT-PCR results indicated that LMAF markedly reduced the LPS-elevated expression of *Cxcl2*, *Tnf*, *Ccl2*, *Il1b*, and *Csf2*. Moreover, LMAF effectively increased the proteins expression of Claudin-1, Occludin, and Zonula occluden-1 in Caco-2 cells. This study highlights the potential of LMAF to improve inflammation and intestinal barrier integrity, offering a foundation for further application of low-Mw fucoidan hydrolysates.

## 1. Introduction

Fucoidan, a sulfated polysaccharide with rich content of fucose, is sourced from brown algae and marine invertebrates [1]. The structurally complex polysaccharide has a backbone composed of repeating α-(1→3)-linked l-fucopyranose residues or an alternating combination of α-(1→3) and α-(1→4)-linked l-fucopyranose residues. Varied amounts of xylose, galactose, mannose, glucose, arabinose, and uronic acids are shown in fucoidan [2]. Additionally, the C-2, C-4, or, in some cases, C-3 positions of the fucosyl residues are substituted by sulfate and/or acetate groups, further contributing to the structural diversity of fucoidan [3,4]. Recognized for its physiological benefits and functional properties, fucoidan has become prominent in food, cosmetics, and pharmaceuticals fields. The diverse functions of fucoidan, particularly as dietary additives, are closely related to its structurally complex features. Fucoidan exhibits a wide range of bioactivities, including anti-inflammatory, anticancer, immune-enhancing, gut barrier support, and modulation of intestinal microbiota balance in the mucosa [2]. Variations in structure, such as molecular weight (Mw), monosaccharide composition, glycosidic bond, and linkage sites, have gained attention due to their impact on microstructure, solubility, bioactivities, and potential applications of fucoidan [5,6]. Moreover, the production of sulfated oligosaccharides and low-Mw hydrolysates from fucoidan via acid or enzymatic hydrolysis has been new interest, given their enhanced solubility and health benefits compared to fucoidan [1].

Inflammation is characterized by the primary reaction of immune system to potentially harmful stimuli, including infection and injury [7]. Diverse stimuli, including bacterial endotoxin lipopolysaccharide (LPS) and other exogenous antigens, can stimulate macrophages and neutrophils to elevate the production and release of inflammatory mediators like nitric oxide (NO), tumor necrosis factor-α (TNF-α), interleukin-1β (IL-1β), and interleukin-6 (IL-6), thereby elevating the risk of chronic inflammation [8,9]. The increased expression of cytokines, chemokines, nuclear factor-κB (NF-κB), and mitogen-activated protein kinases (MAPKs) links inflammatory responses with the development of diseases such as cancer, colitis, diabetes, and obesity [10,11]. Controlling excessive inflammatory responses is crucial for the balance of the immune system and overall health [11]. In recent years, fucoidan extracted from algae has been extensively studied for its various biological activities, including antitumor, immunomodulatory, and antiviral effects, with particular focus on its regulatory role in inflammatory diseases such as colitis [1,12]. Fucoidan has the ability to block lymphocyte adhesion and invasion, inhibit inflammatory-related enzymes, and decrease pro-inflammatory cytokine production by downregulating the MAPK and NF-κB signaling pathways [2]. Despite the growing number of studies on the anti-inflammatory effects of fucoidan, further research is necessary to elucidate the relationship between its polysaccharide structure and its anti-inflammatory and immunomodulatory activities [6,9].

The integrity of the gut barrier is essential for preserving intestinal homeostasis and the overall health of the host. The intestinal barrier prevents pathogens and toxins from entering the bloods and other tissues from the intestinal lumen, thereby maintaining intestinal integrity and immune homeostasis [13]. Gut barrier damage leads to the promotion of inflammatory responses with translocation of microorganisms and endotoxins through the mucosal barrier [14]. The gut barrier is composed of the mucus layer, intestinal epithelial cells, the lamina propria, and microbiota. Intestinal epithelial cells serve as a physical barrier which play an important role in preventing leakage from the intestinal lumen [15]. Tight junctions between intestinal epithelial cells, composed of transmembrane proteins such as Zonula occluden (ZO), Claudin, and Occludin, also play a critical role in the integrity of the intestinal epithelial paracellular barrier and prevent luminal antigens or bacteria from permeating the mucosa [16]. Research indicates that dietary polysaccharides, commonly found in natural foods, significantly influence the intestinal barrier function [17]. Identifying natural compounds that regulate intestinal barrier function and uncovering the associated modulation mechanisms is of importance. Fucoidan has been reported to improve intestinal barrier function by increasing the expression of tight junction proteins [18]. A previous study demonstrated the protective effects of fucoidan derived from the sea cucumber *Acaudina molpadioides* and its enzymatic hydrolysates, with varying Mws (50–500 kDa), in a mouse model of cyclophosphamide-induced intestinal mucositis [19]. Fucoidan and its enzymatic hydrolysates protected the intestinal mucosa from injury and enhanced the expression of IL-6 and IL-10, whereas differing effects of the varied Mw of fucoidan were observed. Given that low-Mw hydrolysates and derived oligosaccharides of fucoidan may exhibit different functional activities due to structural variations, further research is required to explore the protective effects of fucoidans with different Mw on intestinal barrier function.

Several experimental models have been designed to investigate the anti-inflammation and intestinal barrier-improving functions of natural products. Caco-2 cells are a human colon carcinoma cell line commonly used to simulate the characteristics and functions of the human small intestinal epithelial cell layer, capable of producing tight junctions, microvilli, and transport proteins specific to intestinal epithelial cells [20]. In culture, Caco-2 cells can develop a monolayer structure that resembles the structure and function of small intestinal epithelial cells [21]. Due to their stability and reproducibility, Caco-2 cells have been widely used in gut-related research. It is noteworthy that Caco-2 cells alone do not adequately represent the cell interactions occurring in the intestinal environment in vivo. Studies have utilized co-culture models of intestinal epithelial and immune cells to reflect physiological cell interactions through in vitro settings [22]. RAW264.7 cells are widely used as an in vitro model for screening anti-inflammatory and immunomodulatory substances [23]. The co-culture of Caco-2 and RAW264.7 cells enable the assessment of substances that improve intestinal barrier function by modulating the interaction between immune cells and intestinal epithelial cells, thus exerting anti-inflammatory and immunomodulatory effects. Transepithelial electrical resistance (TEER) is a widely used method to assess the integrity of epithelial cell monolayer barriers [24]. Inflammatory response can be induced in RAW264.7 cells when stimulated by inflammatory factors, releasing inflammatory mediators such as NO, TNF-α, IL-1β, and IL-6, which are involved in inflammatory diseases [25]. Consequently, the inflammatory state of the co-culture model can be evaluated by measuring TEER, NO, and cytokine levels.

In this study, fucoidan extracted from *Laminaria japonica* was used to obtain degradation products of different Mw through acid hydrolysis and ethanol precipitation. The effect of fucoidan, low-Mw acidolyzed fucoidan (LMAF, <1.5 kDa), and high-Mw acidolyzed fucoidan (HMAF, 1.5–20 kDa) on inflammatory and intestinal barrier dysfunction were investigated in vitro through LPS-stimulated Caco-2 and RAW264.7 co-culture models. TEER, NO production, and pro-inflammatory cytokines were measured after fucoidan, LMAF, and HMAF treatment. Transcriptomic analysis and real-time quantitative polymerase chain reaction (RT-PCR) were performed to reveal the significantly regulated genes in enriched pathways of RAW264.7 cells in co-cultures. The enhancement of the intestinal cell barrier function in Caco-2 cells was measured using immunofluorescence. This study demonstrates the potential of LMAF to improve inflammation and intestinal barrier integrity, providing a basis for future research on the application of fucoidans and their low-Mw hydrolysates.

## 2. Materials and Methods

### 2.1. Materials

*Laminaria japonica* was harvested from kelp farms in Rongcheng, Shandong, China, in March 2021. The algae samples were sun-dried and stored at −20 °C. Dulbecco’s modified Eagle medium (DMEM), trypsin-EDTA digest, non-essential amino acids, penicillin mix, L-glutamine, penicillin-streptomycin, and PBS buffer were purchased from Solarbio Science & Technology Co., Ltd. (Beijing, China). Certified fetal bovine serum (FBS) was procured from Biological Industries (Kibbutz Beit Haemek, Israel). LPS was purchased from Sigma (Saint Louis, MO, USA). Cell Counting Kit-8 (CCK8) was obtained from APExBio Technology Co., Ltd. (Houston, TX, USA). The NO assay kit was purchased from Beyotime Biotechnology Co. Ltd. (Shanghai, China). TNF-α, IL-1β, IL-6, and IL10 ELISA kits were acquired from Elabscience Biotechnology Co., Ltd. (Wuhan, China). ZO-1 Rabbit Polyclonal Antibody, along with other antibodies, were sourced from Biyuntian Biotechnology Co., Ltd. (Shanghai, China). All other chemicals and reagents were analytical grade and were purchased from Sinopharm Chemical Reagent Co., Ltd. (Shanghai, China).

### 2.2. Preparation of Fucoidan Hydrolysates with Different Molecular Weight

Fucoidan was acquired according to methods described in previously established study [26]. Fucoidan was extracted from dried *Laminaria japonica* through hot water extraction, followed by anion-exchange chromatography and ethanol precipitation. The extracted fucoidan had an average Mw of 654.55 kDa and was subsequently acidolyzed with trifluoroacetic acid. Acidolyzed fucoidan was separated into low-molecular-weight (LMAF, <1.5 kDa) and high-molecular-weight fractions (HMAF, 1.5–20 kDa) via ethanol precipitation, centrifugation, and freeze-drying, as described in our previous study [27]. Fucoidan, LMAF, and HMAF exhibited sulfate contents of 33.68%, 30.51%, and 26.03% of total dry mass, respectively. Fucoidan and LMAF contained uronic acid contents of 9.39% and 7.33% of total dry mass, whereas HMAF had a significantly higher uronic acid content of 22.89%. According to the monosaccharide composition from our previous study, LMAF showed the highest fucose content at 62.83 mol%, followed by fucoidan at 55.55 mol%. HMAF had the lowest fucose content at 14.62 mol%, but the highest glucuronic acid content at 42.49 mol%.

### 2.3. Cell Culture

Intestinal epithelial cell line Caco-2 cells and murine macrophage cell line RAW264.7 cells were sourced from the cell bank of the type culture collection of the Chinese Academy of Science (Shanghai, China). Caco-2 cells were cultured in DMEM enriched with 16% (*v*/*v*) FBS, 1% (*v*/*v*) non-essential amino acids, and 1% (*v*/*v*) l-glutamine. RAW264.7 cells were cultured in DMEM supplemented with 10% (*v*/*v*) FBS. Both cell types were maintained in a humidified incubator with 5% CO_2_ at 37 °C. Logarithmically growing cells were used for subsequent experiments.

### 2.4. Determination of Cell Viability

To identify the concentration of fucoidan hydrolysates with different structures, cells were treated with a range of concentrations (100 μg/mL, 300 μg/mL, 500 μg/mL, 1 mg/mL, 5 mg/mL, and 10 mg/mL) of fucoidan, LMAF, and HMAF in a 96-well plate for 24 h, followed by CCK8 cell viability testing. Similarly, varying concentrations of fucoidan and its hydrolysates were added with LPS (1 μg/mL) and incubated for 24 h. Cell cultures were mixed with the CCK-8 solution at a 9:1 volume ratio and incubated for 30 min following the CCK-8 kit instructions. The absorbance value for each treatment was measured at 450 nm wavelength. The concentration yielding a cell survival rate above 90% was selected for subsequent experiments. The formulas for calculating survival rate of fucoidan hydrolysates on cells were as follows:

Cell survival rate (%) = (Ae − Ab)/(Ac − Ab) × 100

where Ae represents the absorbance value of the experimental group, Ac represents the absorbance value of the control group, and Ab represents the absorbance value of the blank group.

### 2.5. Caco-2/RAW264.7 Co-Culture Model

A Caco-2/RAW264.7 co-culture model was established with slight modifications according to previously published method [28]. Caco-2 cells were plated on transwell insert plates (Corning Costar Corp., Cambridge, MA, USA) at 1.5 × 10^5^ cells/cm^2^ and cultured for 21 days to form a monolayer with a TEER value over 400 Ω·cm^2^, measured with a Millicell ERS-2 volt-ohm meter (Millipore Corporation, Billerica, MA, USA). DMEM was changed every other day for the first week and daily for the next 14 days. RAW264.7 macrophages were plated on 12-well plates at 2 × 10^5^ cells/well and adhered overnight. On day 21, transwell inserts with Caco-2 cells were added to the 12-well plates containing RAW264.7 macrophages. LPS (1 μg/mL) was added to each basolateral side of the co-culture model. Fucoidan, HMAF, and LMAF were applied to the apical side of the transwell insert, respectively. To investigate the anti-inflammatory properties of fucoidan hydrolysates, co-culture cells were treated for 24 h with LPS (1 μg/mL), Fucoidan (300 μg/mL) + LPS (1 μg/mL), LMAF (300 μg/mL) + LPS (1 μg/mL), and HMAF (300 μg/mL) + LPS (1 μg/mL). Co-culture cells with only DMEM medium served as the control group. After incubation, cell supernatants from the basolateral side were collected to measure TNF-α, IL-1β, IL-10, IL-6, and NO levels following kit manufacturers. Cells were collected for transcriptomic analysis.

### 2.6. RNA Extraction and Transcriptomic Analysis

Total RNA of the RAW264.7 cells collected from the co-culture model was extracted using TRIzol^®^ Reagent (Life Technologies, Inc., Carlsbad, CA, USA) as per the manufacturer’s instructions. Sequencing utilized high-quality RNA selected based on determination and quantification with Agilent 5300 Bioanalyser (Agilent Technologies, Palo Alto, CA, USA) and NanoDrop 2000 (Thermo Fisher Scientific Inc., Waltham, MA, USA). RNA purification, reverse transcription, library construction, and sequencing were conducted at Shanghai Majorbio Bio-pharm Biotechnology Co., Ltd. (Shanghai, China) according to Illumina’s instructions (Illumina, San Diego, CA, USA). The Illumina^®^ Stranded mRNA Preparation Kit (Illumina, San Diego, CA, USA) was used to construct a paired-end RNA-seq library (300 bp) on a NovaSeq Xplus sequencer (Illumina, San Diego, CA, USA). Fastp (https://github.com/OpenGene/fastp, accessed on 1 March 2024, version 0.19.5) was used to trim and quality-control raw reads. The clean reads were aligned to the reference genome using HISAT2 (https://daehwankimlab.github.io/hisat-genotype/, accessed on 1 March 2024, version 2.1.0) and assembled with StringTie (https://github.com/gpertea/stringtie, accessed on 1 March 2024, version 2.1.2).

Transcript expression level was calculated based on transcripts per million reads (TPM). Gene abundances were quantified using RSEM (https://deweylab.github.io/RSEM/, accessed on 1 March 2024, version 1.3.3), and differentially expressed genes (DEGs) were identified using DESeq2 (https://bioconductor.org/packages/release/bioc/html/DESeq2.html, accessed on 1 March 2024, version 1.24.0) [29,30]. DEGs between LPS and normal control group were filtered with criteria of fold change (FC) ≥1.5 and corrected *p* value ≤ 0.05. DEGs between LMAF and LPS group were identified using a filter of FC ≥1.5 and *p* value ≤ 0.05. DEGs were annotated for Biological Process (BP), Cellular Component (CC), and Molecular Function (MF) using the GO database and for pathways using the KEGG database with Diamond software (https://www.crystalimpact.com/diamond/Default.htm, accessed on 1 March 2024, version 0.9.24). GO and KEGG database were utilized to annotate which DEGs were significantly enriched at a Benjamini and Hochberg (BH)-corrected *p* value rate of ≤ 0.05 by Goatools (https://github.com/tanghaibao/goatools, accessed on 1 March 2024, version 0.6.5) and KOBAS (http://bioinfo.org/kobas, accessed on 1 March 2024, version 2.1.1), respectively [31]. The relationships among samples were analyzed through principal component analysis (PCA). Co-expressed and specifically expressed genes among DEGS sets were obtained through Venn analysis. Protein interaction network analysis was conducted based on STRING database (https://cn.string-db.org/, accessed on 1 March 2024) and visualized through Python on the online tool of Majorbio Cloud Platform (https://cloud.majorbio.com/page/tools/, accessed on 1 March 2024, accessed on 1 March 2024).

### 2.7. Real-Time Quantitative PCR of Key Genes

RT-PCR was applied to assess the expression of inflammation-associated genes at the mRNA level. Total RNA extraction from three cell samples per group was achieved using Takara RNAiso Plus kits (Takara Biomedical Technology Co., Ltd., Beijing, China). Reverse transcription was performed by PrimeScript™ RT Master Mix Kit (Takara Biomedical Technology Co., Ltd., Beijing, China). RT-PCR was performed using TB Green Premix Ex Taq II on the QuantStudio 7 Flex Real-Time PCR system (Thermo Fisher Scientific, Waltham, MA, USA). The relative mRNA expression was normalized to β-actin and calculated using 2^−ΔΔCt^. Primers used for RT-PCR were designed based previous studies and listed in Supplementary Table S1 [32,33,34]. Primers sequences were sourced from Sangon Biotech Co., Ltd. (Shanghai, China).

### 2.8. Immunofluorescence of Caco-2 Cells

Caco-2 cells (2 × 10^5^ cells/well) were seeded in 12-well plates and exposed to fucoidan, LMAF, and HMAF at a concentration of 300 μg/mL. After cultivation for 24 h, cells were washed with PBS and fixed with 4% paraformaldehyde for 15 min. Cells were then permeabilized using 0.5% Triton X-100 for 10 min and incubated with 10% anti-goat serum at 37 °C for 30 min. Subsequently, cells were stained overnight at 4 °C with ZO-1 rabbit polyclonal antibody, Occludin rabbit polyclonal antibody, and Claudin-1 rabbit polvclonal antibody, respectively, each diluted 1:100 in blocking buffer. Following this incubation, cells were treated with Alexa Fluor 488 (Thermo Fisher Scientific Inc., Waltham, MA, USA) for 1 h. Finally, cells were stained with 4′,6-diamidino-2-phenylindole (DAPI, Thermo Fisher Scientific Inc., Waltham, MA, USA) containing anti-fluorescein. The stained cells were then examined and photographed using an DMi8 inverted fluorescence microscope (Leica, Wetzlar, Germany).

### 2.9. Statistics Analysis

The values are shown as mean ± standard deviation (SD). During cell co-culture experiments, statistical significance between LPS-treated group and other groups was performed using Student’s *t*-test to analyze TEER values, TNF-α, IL-1β, IL-10, IL-6, and NO levels (*n* = 5). RNA-seq data analysis for differentially expressed genes between groups was performed using DESeq2 (*n* = 3). GO and KEGG annotation functional enrichment analyses were carried out using Goatools and KOBAS with the BH method. Relative mRNA expression changes across all groups in RNA-seq analysis were analyzed by one-way ANOVA followed by Tukey’s HSD post hoc test. RT-PCR expression of inflammation amelioration related genes in fucoidan and acidolyzed hydrolysates treated groups were analyzed by one-way ANOVA followed by Tukey’s HSD post hoc test when compared to LPS group (*n* = 3). A *p*-value of less than 0.05 was considered significant. All statistical analyses were performed using SPSS 22.0 software (SPSS, Inc., Chicago, IL, USA).

## 3. Results and Discussion

### 3.1. The Effects of Fucoidan Hydrolysates on Cell Viability

**Figure 1:**
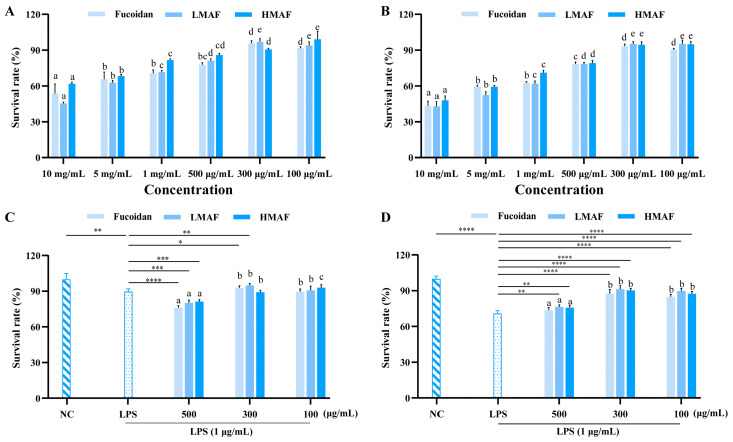
Effects of fucoidan and its hydrolysates on cell viability. Sample toxicity in Caco-2 (**A**) and RAW264.7 (**B**) cells. Protective effect in LPS (1 μg/mL)-induced Caco-2 (**C**) and RAW264.7 (**D**) cells. Cells were treated with fucoidan, LMAF, and HMAF for 24 h, respectively, followed by assessment using the CCK8 testing method. Data are expressed as the mean ± SD (*n* = 5). ^a–e^ Significant differences among varied concentrations for each fucoidan and its hydrolysates were analyzed using one-way ANOVA with a post hoc Tukey’s HSD test (*p* < 0.05). Statistical significance between LPS-treated group and other groups was performed using Student’s *t*-test (* *p* < 0.05, ** *p* < 0.01, *** *p* < 0.001, **** *p* < 0.0001). NC, normal control.

To determine the experimental concentrations of fucoidan, LMAF, and HMAF for Caco-2 and RAW264.7 cells in subsequent experiments, the CCK-8 experiment was used to evaluate the cytotoxic effects of fucoidan and its hydrolysates. The initial concentrations of samples and LPS were chosen according to previously established and published protocols [35,36]. As depicted in Figure 1A,B, concentrations exceeding 500 μg/mL notably reduced cell viability, indicating cytotoxic effects. At treatment concentrations of 300 and 100 μg/mL, fucoidan, LMAF, and HMAF maintained Caco-2 and RAW264.7 cell survival rates above 90%. In both Caco-2 and RAW264.7 cells, LPS stimulation caused a marked reduction in cell viability relative to unstimulated cells. The survival rate of Caco-2 cells dropped to 89.75 ± 2.19% (*p* < 0.01) following LPS treatment in comparison to the normal group (Figure 1C). Fucoidan and its hydrolysates demonstrated cytotoxicity at 500 μg/mL, causing an additional reduction in the survival rate of LPS-stimulated Caco-2 cells. Fucoidan (*p* < 0.05), LMAF (*p* < 0.01), and HMAF at 300 μg/mL enhanced the survival of LPS-stimulated Caco-2 cells more effectively than the 100 μg/mL concentration. LPS caused a more pronounced decline in RAW264.7 cell survival, lowering it to 70.77 ± 2.39% (*p* < 0.0001) compared to the control group (Figure 1D). Fucoidan, LMAF (*p* < 0.01), and HMAF (*p* < 0.01) at 500 μg/mL increased the survival of LPS-stimulated RAW264.7 cells. At concentrations of both 300 μg/mL and 100 μg/mL, fucoidan and its hydrolysates significantly enhanced the survival rate of LPS-stimulated RAW264.7 cells (*p* < 0.0001), with the best effects seen at 300 μg/mL, where fucoidan, LMAF, and HMAF showed survival rates of 87.55 ± 3.36%, 91.21 ± 2.98%, and 90.33 ± 1.51%, respectively. Accordingly, the sample concentration was set to 300 μg/mL in the subsequent experiments.

### 3.2. The Effects of Fucoidan Hydrolysates on Intestinal Epithelial and Cytokines Production in LPS-Stimulated Co-Cultures

**Figure 2:**
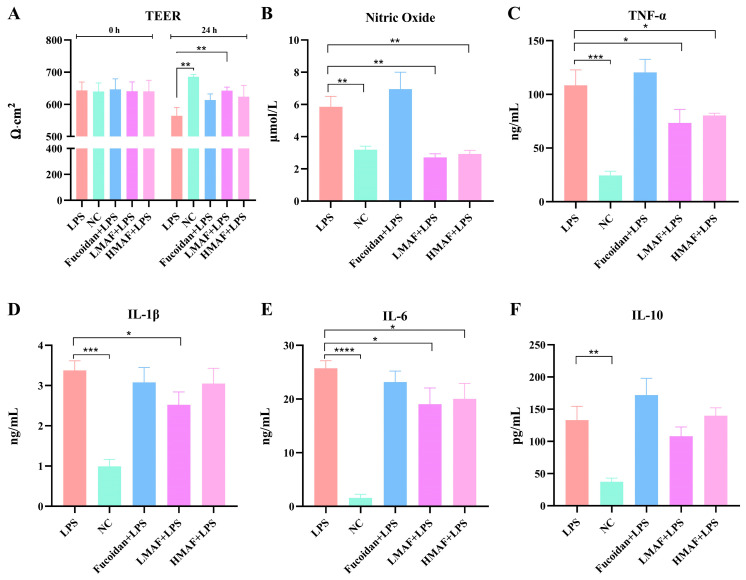
The effect of fucoidan and its acidolyzed hydrolysates on intestinal epithelial integrity and cytokines production in Caco-2/RAW264.7 co-cultures with LPS stimulation. (**A**) Transepithelial electrical resistance (TEER). (**B**) Nitric oxide production. Inflammatory cytokine production of (**C**) TNF-α, (**D**) IL-1β, (**E**) IL-6, and (**F**) IL-10 with treatment of fucoidan, LMAF, and HMAF, respectively. Data are presented as the mean ± SD (*n* = 5). Statistical significance between LPS-treated group and other groups was performed using Student’s *t*-test (* *p* < 0.05, ** *p* < 0.01, *** *p* < 0.001, **** *p* < 0.0001). NC, normal control.

TEER values were measured in co-cultures of Caco-2 and RAW264.7 cells treated with fucoidan, LMAF, and HMAF to assess the integrity of intestinal epithelial monolayer (Figure 2A). The initial TEER values of each group were between 640 and 647, indicating the integrity of the Caco-2 cell monolayer model. An increase in TEER was noted in the normal control group (NC), with TEER value increasing from 640.67 ± 26.84 to 686.67 ± 6.81. LPS caused a significant decrease compared to NC (*p* < 0.01), with TEER value decreasing from 643.33 ± 26.03 to 564.00 ± 26.29. TEER values displayed a downward trend in all groups treated with LPS. Treatment with fucoidan, LMAF, and HMAF led to an increase in TEER compared to the LPS group, reaching 613.33 ± 18.90, 642.33 ± 11.15 (*p* < 0.01), and 624.67 ± 35.23, respectively. The bioactivity of fucoidan is associated with its complex structure, especially the degree of polymerization and sulfate content [1]. The Mw of functional carbohydrates has been linked to immunomodulatory activity, where an excessively high Mw may suppress immune responses [37]. Hydrolysis is recognized as a considerable strategy for improving the bioactivity of high-Mw carbohydrates. For instance, acid hydrolysates exhibited enhanced immunomodulatory activity in RAW264.7 cells compared to native dextran, possibly due to the lower-Mw hydrolysates binding more efficiently to cell receptors [38]. In this study, the low-Mw and considerably high sulfate content of LMAF were thought to contribute to its efficacy in restoring the integrity of LPS-damaged intestinal epithelial monolayers, in comparison to the original fucoidan and HMAF.

RAW264.7 cells serve as a commonly used in vitro model for assessing the anti-inflammatory and immunomodulatory properties of natural substances. When stimulated by inflammatory agents, particularly LPS, RAW264.7 cells can generate a strong inflammatory response, activating nuclear transcription factors such as NF-κB, leading to the production of inflammatory mediators including NO, TNF-α, IL-1β, and IL-6 [25,39]. NO is a short-lived bioactive molecule released into endothelial cells, actively participating in inflammatory disorders. Excessive NO production by activated macrophages has been linked to various inflammatory diseases [40]. LPS stimulation led to a significant increase in NO release compared to NC group (3.19 ± 0.23 μmol/L), obtaining 5.86 ± 0.65 μmol/L (*p* < 0.01, Figure 2B). Both LMAF (2.72 ± 0.22 μmol/L) and HMAF (2.93 ± 0.23 μmol/L) induced a significant decrease in NO levels compared to the LPS group (*p* < 0.01). LMAF exhibited the most pronounced effect, resulting in a significant reduction in the release of NO compared to the LPS. However, a higher NO level was observed with fucoidan treatment. The immunomodulatory activity is a major focus of fucoidan research. It is widely believed that the fucoidan’s main protective mechanism is the activation of the host immune response. Research has indicated that low-Mw fucoidan exhibits anti-inflammatory activity, while high-Mw fucoidan shows immunomodulatory activity [41]. Macrophages, recently recognized for their potential in cancer immunotherapy, eliminate tumor cells via phagocytosis and release tumor-suppressing molecules such as NO, TNF-α, IL-1β, and IL-6, which play a crucial role in the host immune defense system [42]. It has been reported that fucoidan modulated the MAPK and NF-κB signaling pathways and enhanced NO release in RAW264.7 macrophages [43]. Likewise, fucoidan prepared from *Undaria pinnatifida* efficiently induced NO release in RAW264.7 macrophages and stimulated TNF-α and IL-6 secretion [44]. The link between fucoidan’s structure and its role in immunomodulation remains controversial. Given that the immune-stimulating and anti-inflammatory properties of fucoidan have emerged as a key research focus in the functional food field, its structure–activity relationship requires further investigation.

The production of cytokines, including TNF-α (Figure 2C), IL-1β (Figure 2D), IL-6 (Figure 2E), and IL-10 (Figure 2F) of LPS-stimulated RAW264.7 macrophages, were assessed using ELISA kits in the co-culture model to investigate the anti-inflammatory effects of fucoidan and hydrolysates. LPS significantly increased the secretion of TNF-α (*p* < 0.001), which obtained 108.45 ± 14.30 ng/mL compared to non-stimulated cells (24.42 ± 4.07 ng/mL). Both LMAF (73.46 ± 12.56 ng/mL) and HMAF (80.37 ± 2.10 ng/mL) treatment significantly reduced the secretion of TNF-α compared to LPS group (*p* < 0.05). LPS significantly increased secretion of IL-1β (*p* < 0.001) and IL-6 (*p* < 0.0001) compared to the NC group. Both LMAF (19.03 ± 3.03 ng/mL) and HMAF (20.03 ± 2.88 ng/mL) treatment significantly reduced the secretion of IL-6 cytokines by 26.04% and 22.15%, respectively, compared to LPS group (25.73 ± 1.41 ng/mL) (*p* < 0.05). However, only LMAF (2.52 ± 0.32 ng/mL) significantly reduced the secretion of IL-1β cytokines compared to LPS group (3.37 ± 0.24 ng/mL) (*p* < 0.05). Increased secretion of IL-10 was shown in LPS compared to the NC group (*p* < 0.01), while LMAF reduced the secretion of IL-10 cytokines. However, no significant increase was observed with fucoidan treatment. Fucoidan showed slight reduction in IL-1β and IL-6 compared to LPS group, along with an increase in both TNF-α and IL-10.

Previous studies have reported the anti-inflammatory activity of fucoidan derived from various algal sources. Fucoidan isolated from fermented *Sargassum fusiforme* demonstrated its effects on inhibiting NO production induced by LPS in RAW264.7 cells, as well as TNF-α, IL-1β, and IL-6 [45]. Low-Mw fucoidan from *Sargassum hemiphyllum* showed bioactivity to decrease IL-1β and TNF-α secretion while enhancing the immune function of LPS-exposed Caco-2 cells [46]. Additionally, low-Mw fucoidan extracted from *Saccharina japonica* improved inflammatory responses in atherosclerotic mice as well as downregulating IL-6 and upregulating IL-10 transcription levels [47]. The structure and biological activity of fucoidan are influenced by factors like seaweed species, growth region, harvest season, and extraction and processing methods [48,49]. The anti-inflammatory properties of fucoidan appear to be closely associated with the seaweed type, monosaccharide composition, and sulfate content [9]. Further research is required to develop low-Mw fucoidan hydrolysates and to clarify the impact of structural differences on their anti-inflammatory properties.

### 3.3. Effects of LMAF on Transcriptome of RAW264.7 Cells in Co-Cultures

**Figure 3:**
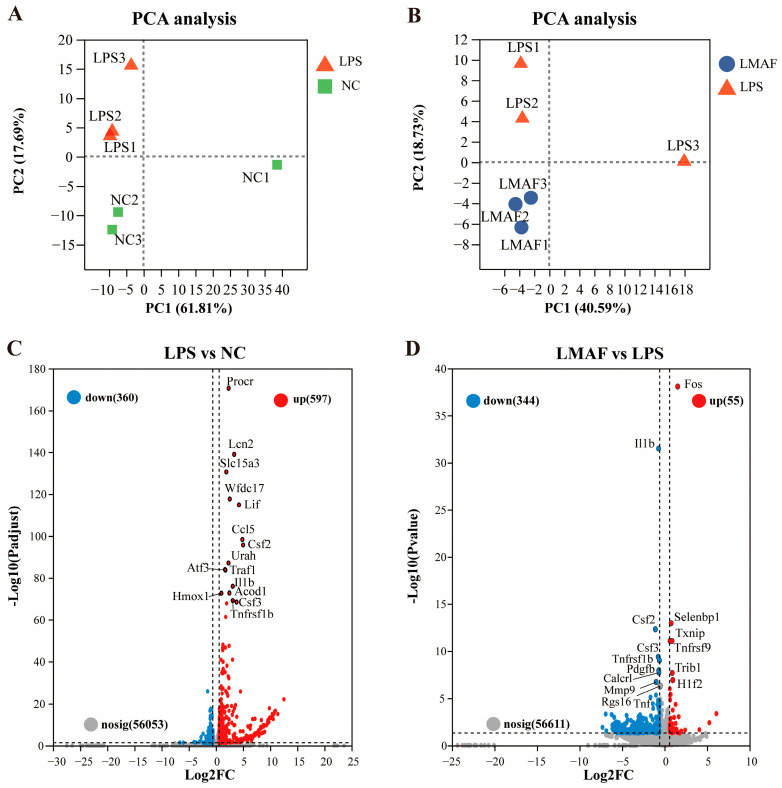
Impact of LMAF on the transcriptome of RAW264.7 cells in co-cultures. (**A**) PCA plot of LPS versus the normal control (NC) group. (**B**) PCA plot comparing the LMAF and LPS group. (**C**) Volcano plot highlighting significant DEGs between LPS and NC. (**D**) Volcano plot of significant DEGs between LMAF and LPS. Red dots represent upregulated DEGs; blue dots represent downregulated DEGs (*n* = 3).

In the co-cultures, LMAF demonstrated better effects than HMAF and fucoidan in alleviating LPS-induced intestinal barrier damage, reducing NO, and decreasing the release of inflammatory factors. To further investigate the mechanism by which LMAF alleviates LPS-induced inflammation, RNA-Seq analysis was performed on RAW264.7 cells in co-cultures from NC, LPS, and LMAF group. A total of 40.49–49.01 million clean reads were filtered in each sample with Q30 exceeding 94.24% (Table S2). According to PCA plot, LPS induced transcriptomic changes in RAW264.7 cells, distinguishing all LPS-treated samples from the normal control group (Figure 3A). PC2 filtering separated LPS from NC accounting for 17.69% of total variation. LMAF treatment induced clear differences compared to LPS, with PC2 filtering separated LMAF from LPS, representing 18.73% of total variation (Figure 3B). Differential volcano plot showed that 957 DEGs were identified in LPS compared to NC, with 360 genes downregulated and 597 genes upregulated, respectively (Figure 3C). After 21 days of LMAF treatment, compared with LPS, a total of 399 DEGs were identified in LMAF, indicating that LMAF supplementation induced an effect on modulating transcription, with 344 DEGs downregulated (Figure 3D).

**Figure 4:**
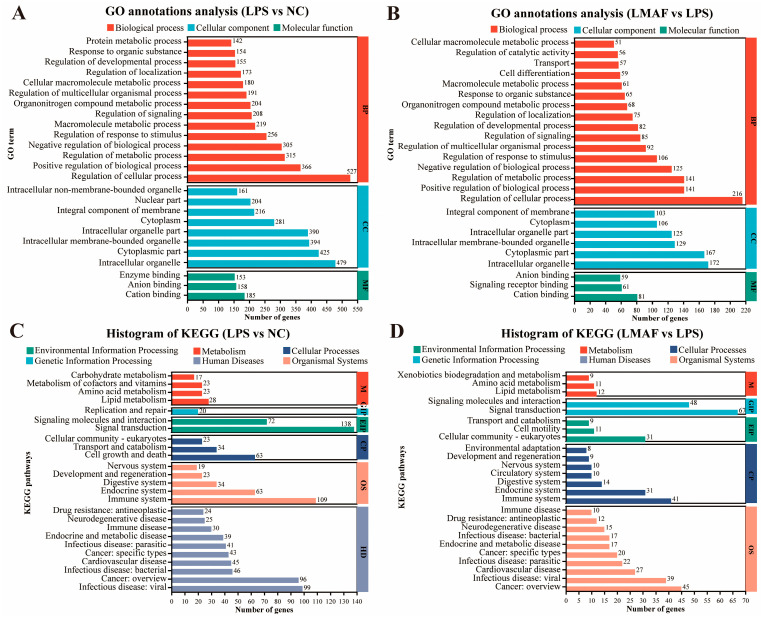
Functional annotation analysis of differentially expressed genes. GO annotations analysis of LPS versus the normal control (NC) group (**A**) and LMAF versus the LPS group (**B**). KEGG annotations analysis of LPS versus the NC group (**C**) and LMAF versus the LPS group (**D**) (*n* = 3).

Upon LPS stimulation, significantly altered genes in RAW264.7 cells were primarily associated with biological processes (BP), including regulation of cellular processes (527 DEGs), positive regulation of biological processes (366 DEGs), and regulation of metabolic processes (315 DEGs). Moreover, altered genes were annotated into cellular component (CC), including intracellular organelle (479 DEGs), cytoplasmic part (425 DEGs), and intracellular membrane-bounded organelle (394 DEGs) (Figure 4A). Likewise, in the co-culture model of LPS-induced inflammation, LMAF intervention primarily influenced genes within the biological process GO terms of regulation of cellular processes (216 DEGs), positive regulation of biological processes (141 DEGs), and regulation of metabolic processes (141 DEGs). Similar to LPS, intracellular organelle (172 DEGs) and cytoplasmic part (167 DEGs) were also GO terms with abundant DEGs annotations (Figure 4B). KEGG annotation analysis identified the key pathways in RAW264.7 cells affected by LPS and LMAF intervention. Under LPS stimulation, the significantly altered genes in RAW264.7 cells were primarily annotated to KEGG pathways such as signal transduction (138 DEGs), immune system (109 DEGs), infectious disease: viral (99 DEGs), and cancer: overview (96 DEGs) (Figure 4C). In the co-culture model of LPS-induced inflammation, LMAF intervention primarily impacted KEGG pathways like signal transduction (67 DEGs) and signaling molecules and interaction (48 DEGs), followed by cancer: overview (45 DEGs) and immune system (41 DEGs) in RAW264.7 cells (Figure 4D).

**Figure 5:**
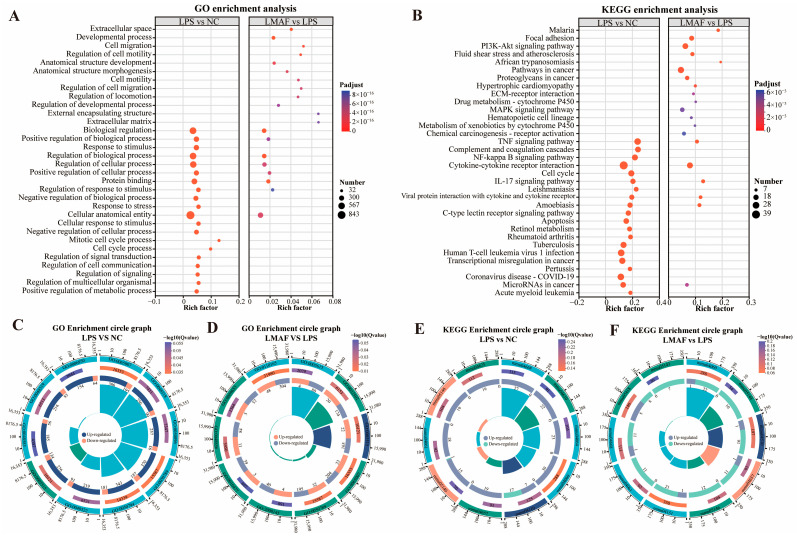
Enrichment analysis of differentially expressed gene. (**A**) GO enrichment analysis. (**B**) KEGG enrichment analysis. Multidimensional GO enrichment circle diagram of LPS versus the NC group (**C**) and LMAF versus the LPS group (**D**). Multidimensional KEGG enrichment circle diagram of LPS versus the NC group (**E**) and LMAF versus the LPS group (**F**) (*n* = 3). NC, normal control.

Further functional enrichment analysis was conducted on the DEGs caused by LPS and LMAF intervention to evaluate the main affected GO terms (Figure 5A) and KEGG pathways (Figure 5B). GO enrichment analysis showed that DEGs induced by LPS intervention were significantly enriched in biological regulation (GO:0065007, 581 DEGs), positive regulation of biological process (GO:0048518, 355 DEGs), response to stimulus (GO:0050896, 350 DEGs), regulation of biological process (GO:0050789, 557 DEGs), and regulation of cellular process (GO:0050794, 524 DEGs) (Figure 5A,C). In RAW264.7 cells with LPS-induced inflammation, DEGs caused by LMAF intervention were significantly enriched in the extracellular space (GO:0005615, 84 DEGs), developmental process (GO:0032502, 144 DEGs), and protein binding (GO:0005515, 187 DEGs) (Figure 5A,D). Notably, DEGs induced by both LPS and LMAF intervention were enriched in biological regulation, cellular anatomical entities, and protein binding, suggesting that LMAF treatment may mitigate inflammation by affecting genes linked to these GO terms (Figure 5A).

KEGG functional enrichment analysis showed that DEGs caused by LPS intervention were significantly enriched in the TNF signaling pathway (mmu04668, 27 DEGs), complement and coagulation cascades (mmu04610, 22 DEGs), NF-kappa B signaling pathway (mmu04064, 23 DEGs), cytokine cytokine receptor interaction (mmu04060, 39 DEGs), and cell cycle (mmu04110, 24 DEGs) (Figure 5B,E). In RAW264.7 cells with LPS-induced inflammation, LMAF intervention caused significant enrichment of DEGs in cytokine–cytokine receptor interaction (mmu04060, 23 DEGs), focal adhesion (mmu04510, 17 DEGs), and the IL-17 signaling pathway (mmu04657, 12 DEG) (Figure 5B,F). It was noteworthy that the DEGs induced by LPS and LMAF intervention were enriched in the TNF signaling pathway, cytokine–cytokine receptor interaction, and IL-17 signaling pathway, indicating that LMAF may alleviate inflammation levels by regulating key genes in inflammatory factor-related KEGG pathways (Figure 5B). TNF-α (TNF) acts as a crucial regulator of immune responses in both healthy organisms and diseases states, exerting various functions in many diseases by mediating signaling for cell survival and apoptosis, particularly in autoimmune and inflammatory diseases [50]. TNF-α, primarily produced by activated macrophages and T cells, signals through TNF receptor 1 (TNFR1) and TNF receptor 2 (TNFR2) [51]. The TNFR1 receptor can activate the transcription factor NF-κB, p38 MAP kinases, and protein kinase JNK. Signaling via TNFR2 is also capable of activating MAP kinases and NF-κB activation [50,52]. Transcriptome analysis in this study indicated that LPS stimulation primarily influenced TNF-related metabolic pathways, and similarly, LMAF mitigated inflammatory symptoms mainly by regulating TNF and cytokine-related pathways. The regulatory effect of LMAF on the TNF signaling pathway requires further investigation.

**Figure 6:**
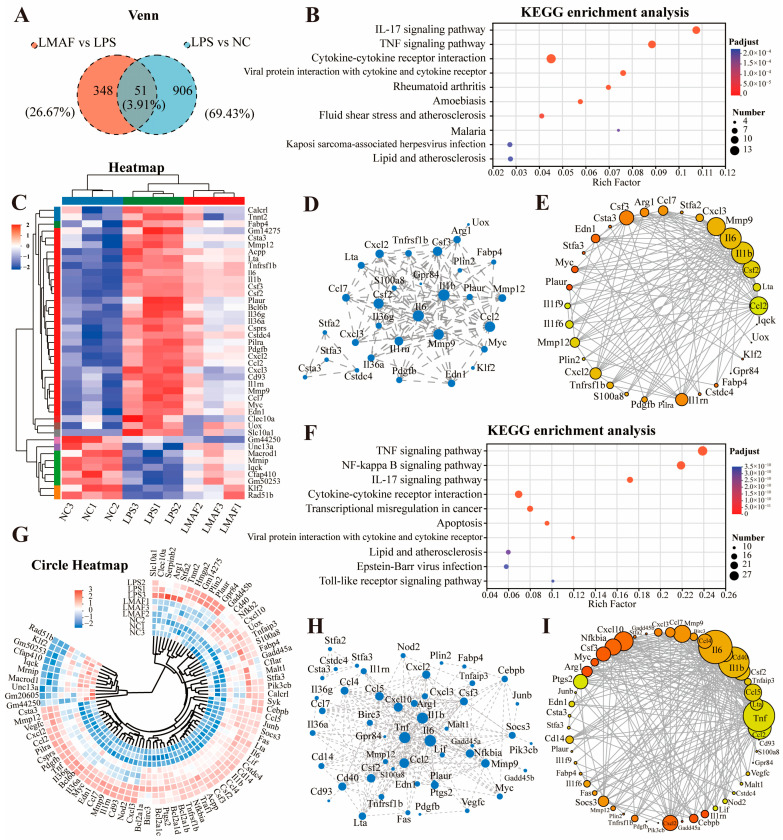
Analysis of key differentially expressed gene. (**A**) Venn diagram of shared DEGs expressed in two libraries of LPS versus the NC group and LMAF versus the LPS group. (**B**) KEGG enrichment analysis of shared DEGs. (**C**) Heatmap plot of shared DEGs. The red and blue colors indicate the upregulated and downregulated DEGs, respectively. (**D**) The protein–protein interaction network of shared DEGs. Increasing node indicates a stronger core. (**E**) The gene–gene interaction network among the shared DEGs. (**F**) KEGG enrichment analysis of DEGs expressed in shared enriched KEGG pathway in two libraries of LPS versus the NC group and LMAF versus the LPS group. Circle heatmap plot (**G**), protein–protein interaction network (**H**), and gene–gene interaction network (**I**) of DEGs expressed in shared enriched KEGG pathway. NC, normal control.

To investigate the main metabolic pathways by which LMAF regulates RAW264.7 cells with LPS-induced inflammation, a Venn analysis was performed, comparing shared DEGs between LPS vs. NC and LMAF vs. LPS, resulting in the identification of 51 shared DEGs (Figure 6A). KEGG enrichment analysis of shared DEGs revealed that the DEGs were primarily annotated in the inflammation-related pathways, including cytokine–cytokine receptor interaction, the TNF signaling pathway, and the IL-17 signaling pathway (Figure 6B). Heatmap analysis revealed that LPS markedly elevated the expression levels of inflammation-related genes such as *Tnfrsf1b*, *Il6*, *Il1b*, *Il36g*, *Il36a*, and *Il1rn* (Figure 6C). Additionally, LPS significantly upregulated the expression levels of macrophage inflammation-related genes, including *Csf2*, *Csf3*, *Cxcl2*, *Cxcl3*, *Ccl2*, *Ccl7*, *Mmp9*, *Mmp12*, *Clec10a*, and *Ptgs2*. Protein interaction network analysis demonstrated that the interactions within the biological system underscore the pivotal roles of *Il6*, *Il1b*, *Mmp9*, *Ccl2*, and *Csf2* among the shared DEGs (Figure 6D). The gene–gene interaction network of overlapping DEGs between LPS vs. NC and LMAF vs. LPS further emphasized the critical roles of *Mmp9*, *Il6*, *Il1b*, *Csf2*, and *Ccl2* (Figure 6E).

Further analysis of DEGs in key enriched KEGG pathways involved annotating the DEGs related to the TNF signaling pathway, NF-kappa B signaling pathway, cytokine–cytokine receptor interaction, and IL-17 signaling pathway (Figure 6F). Circle heatmap analysis showed that LPS markedly elevated the expression levels of *Serpinb2*, *Cd40*, *Nfkb2*, *Tnfaip3*, *Cebpb*, *Junb*, *Socs3*, *Tnf*, *Il6*, *Cd14*, *Il1b*, *Nfkbia*, *Tnfrsf1b*, *Il1rn*, and *Il36a* in the inflammation-related pathways (Figure 6G). Protein interaction network analysis (Figure 6H) and gene–gene interaction network analysis (Figure 6I) demonstrated that within the TNF signaling pathway, NF-kappa B signaling pathway, cytokine–cytokine receptor interaction, and IL-17 signaling pathway, LMAF treatment modulated inflammatory responses induced by LPS through influencing the expression levels of key genes such as *Il6*, *Il1b*, *Tnf*, *Ccl2*, *Mmp9*, and *Csf2*.

### 3.4. Effects of LMAF on Expression of Inflammatory Related Genes and Tight Junction Proteins

**Figure 7:**
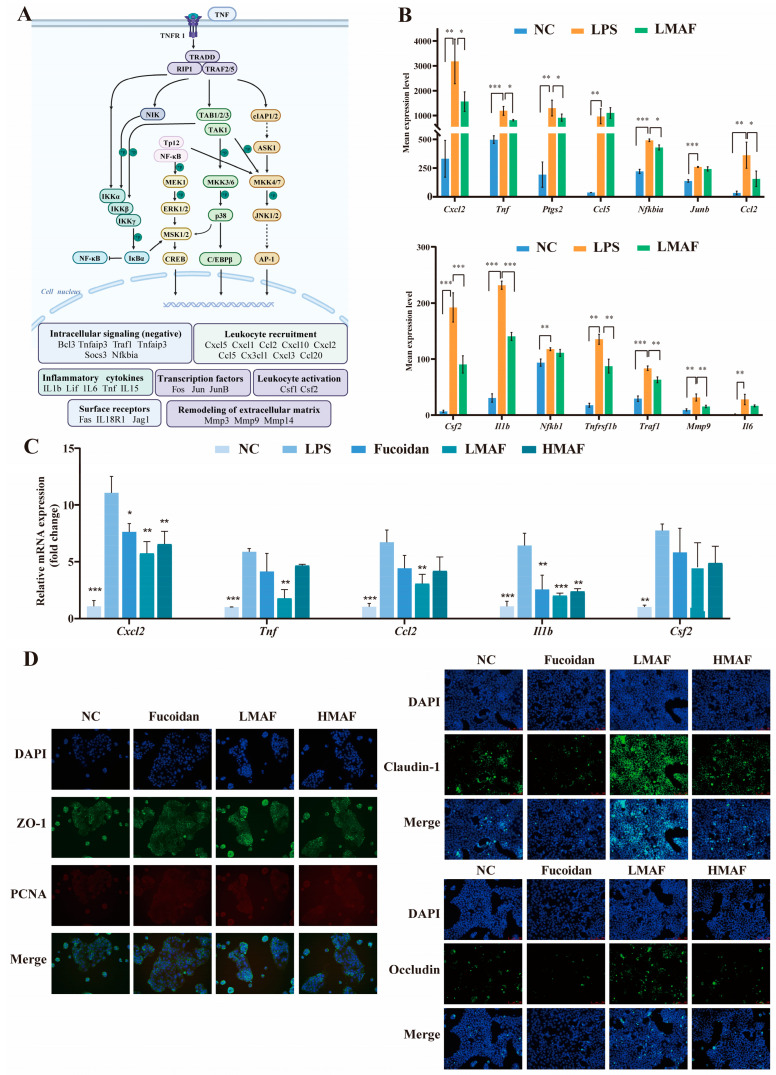
Effects of LMAF on modulating inflammation and intestinal barrier dysfunction in LPS-stimulated Caco-2 and RAW264.7 co-cultures. (**A**) Possible mechanisms of LMAF on ameliorating inflammation in RAW264.7 cells. (**B**) Experssion levels of transcripts in TNF signaling pathway involved in ameliorating inflammation (*n* = 3). Relative mRNA expression changes across all groups in RNA-seq analysis were analyzed by one-way ANOVA followed by Tukey’s HSD post hoc test (* *p* < 0.05, ** *p* < 0.01, *** *p* < 0.001). (**C**) The effects of fucoidan and acidolyzed hydrolysates on RT-PCR expression of inflammatory related genes (*n* = 3). Significant differences of each other groups compared to LPS group were analyzed by one-way ANOVA followed by Tukey’s HSD post hoc test (* *p* < 0.05, ** *p* < 0.01, *** *p* < 0.001). (**D**) The effects of fucoidan and acidolyzed hydrolysates on expression of Claudin-1, Occludin, ZO-1, and proliferating cell nuclear antigen (PCNA) in Caco-2 cells. Protein levels (green) of ZO-1, Occludin, Claudin-1, and PCNA (red) were detected by immunofluorescence. Nuclei were counterstained with DAPI. Scale bar = 100 µm.

Transcriptome analysis revealed that LMAF primarily regulated the LPS-induced inflammatory response by modulating inflammation-related pathways, with key differential genes predominantly enriched in the TNF signaling pathway (Figure 7A). As shown in Figure 7A,B, LPS significantly elevated the expression levels of genes such as *Tnf* (*p* < 0.001), *Nfkb1* (*p* < 0.01), *Ptgs2* (*p* < 0.01), and *Tnfrsf1b* (*p* < 0.01) within the pathway. Furthermore, LPS significantly upregulated the expression of genes involved in leukocyte recruitment (*Cxcl2*, *Ccl2*, *Ccl5*), inflammatory cytokines (*Il1b*, *Il6*), intracellular signaling (negative regulation) (*Traf1*, *Nfkbia*), the synthesis of inflammatory mediators (*Ptgs2*), transcription factors (*Junb*), leukocyte activation (*Csf2*), and remodeling of the extracellular matrix (*Mmp9*). TNF plays a pivotal role in orchestrating inflammatory immune responses. Inhibiting TNF binding to TNFR1 and TNFR2 can attenuate the activation of the MAPK and NF-κB pathways, thereby modulating inflammation [53]. Moreover, binding of TNF to TNFR1 can indirectly promote inflammation through inducing cell death, as dying cells release intracellular components that induce the expression of pro-inflammatory genes in adjacent cells. In the intestinal tract, epithelial cell death caused by inflammation compromises the integrity of the intestinal barrier, facilitating microbial infiltration and further inflammation [53,54]. In this study, LMAF significantly reduced TNF expression compared to the LPS group (*p* < 0.05), along with lowering cytokines and transcription factors elevated by TNF signaling, such as *Cxcl2* (*p* < 0.05), *Ptgs2* (*p* < 0.05), *Ccl2* (*p* < 0.05), *Csf2* (*p* < 0.001), *Il1b* (*p* < 0.001), *Nfkb1*, *Tnfrsf1b* (*p* < 0.01), *Traf1* (*p* < 0.01), *Mmp9* (*p* < 0.01), and *Il6* (Figure 7B).

To analyze the effect of fucoidan and acidolyzed hydrolysates on inflammatory states through the TNF metabolic pathway, RT-PCR experiments were performed on the significantly altered differential genes (Figure 7C). The results indicated that LPS significantly elevated the expression levels of *Cxcl2* (*p* < 0.001), *Tnf* (*p* < 0.001), *Ccl2* (*p* < 0.001), *Il1b* (*p* < 0.001), and *Csf2* (*p* < 0.01) in RAW264.7 cells, as compared to the NC group. In comparison with the LPS group, both fucoidan and its hydrolysates decreased the transcription levels of key genes, with LMAF showing the most pronounced effect. Fucoidan, HMAF, and LMAF all reduced the transcription levels of *Tnf*, yet only the intervention with LMAF reached significance (*p* < 0.01). Moreover, fucoidan (*p* < 0.01), HMAF (*p* < 0.01), and LMAF (*p* < 0.001) each significantly decreased the LPS-induced increase in *Il1b* transcription levels. CXCLs, one subfamily of chemokines, are capable of recruiting and directing various types of immune cells; modulating pathological behaviors like tumor growth, invasion, and metastasis; and are intimately linked with both tumors and inflammatory conditions [55,56]. Research has reported that CXCLs regulate inflammation and immune responses by guiding the migration of leukocytes, including macrophages, and asimultaneously activate multiple signaling pathways, such as the NF-κB and JAK/STAT signaling pathways, to influence tumor cell proliferation and invasion, thereby promoting cancer progression [55]. Specifically, CXCL2 is a growth-related oncogene with crucial biological functions in colorectal cancer [57]. Here, fucoidan (*p* < 0.05), HMAF (*p* < 0.01), and LMAF (*p* < 0.01) all significantly decreased the LPS-induced increase in *Cxcl2* expression, with LMAF showing the most significant reduction. Among inflammation proccess, CCL2 plays a crucial role during biological response by inducing monocyte recruitment, stimulating inflammatory responses, and intensifying the local inflammatory microenvironment [58]. In this study, LMAF significantly lowered the transcription levels of *Ccl2* (*p* < 0.01) compared to the LPS group, with fucoidan and HMAF showing a similar reduction trend.

To further validate the effects of fucoidan and its acidolyzed hydrolysates on intestinal barrier function, the expression levels of tight junction proteins were evaluated using immunofluorescence assays in Caco-2 cells (Figure 7D). Immunofluorescence staining was conducted to examine the localization and expression of Claudin-1, Occludin, and ZO-1 in Caco-2 cells with the treatment of fucoidan and acidolyzed hydrolysates. Claudin-1 exhibited a more diffuse distribution pattern, differing from the membrane localization observed with Occludin and ZO-1. A significantly higher overall fluorescence intensity of Claudin-1, Occludin, and ZO-1 in Caco-2 cells was observed with LMAF treatment compared to treatment of fucoidan and HMAF. The preservation of a fully functional intestinal barrier is crucial for overall health, as it serves to prevent tissue damage and reduces the risk of various diseases. Preserving the integrity of the intestinal barrier, which includes both the epithelial barrier and immune defenses, creates a physical barrier that aids nutrient absorption while potential risk factors [13]. Research indicates that changes in the permeability of the intestinal epithelial monolayer are closely linked to alterations in tight junction proteins [59]. Fucoidan treatment has been reported to improve intestinal barrier function through increasing ZO-1, Claudin-1, and Occludin protein expression in NOD mice [18]. Fucoidan from *Laminaria japonica* strengthened colon barrier integrity by upregulating tight junction proteins (ZO-1, Occludin, and Claudin-1) and alleviated colitis via the TLR4/NF-κB pathway in male Kunming mice [60]. Moreover, high-Mw fucoidan from *Undaria pinnatifida* promoted the expression of tight junction proteins in both Caco-2 cells and mouse colon tissues [61]. Considering that LMAF demonstrated superior ability to enhance tight junction protein expression compared to original fucoidan and HMAF, subsequent studies can further focus on the activity of low-Mw fucoidan hydrolysates and oligosaccharides.

Different functional cell models are used in vitro to simulate the intestinal environment, with the Caco-2 model being one of the most commonly employed and well established [62]. Differentiated Caco-2 monolayers are used in vitro to simulate the intestinal tract because of their morphological similarity, enzyme marker expression, and permeability resembling the human small intestine [63]. Co-culture models of differentiated Caco-2 and immune cells can more closely simulate the in vivo microenvironment, addressing the limitations of monolayer cultures in physiological representation, and are widely applied in in vitro intestinal studies on absorption, transport, barrier function, inflammation, and immunomodulation [62,64]. For enhanced in vitro experiments, the co-culture of Caco-2 and RAW264.7 cells can be combined with HT29-MTX, a mucin-secreting cell line, to develop a mucous layer [65]. Specific cell lines, such as TC-7, a Caco-2 variant, express higher levels of CYP3A4, making them more effective for studies on CYP3A4-dependent metabolism. Additionally, approaches such as genetic modification, gene knockout, and subcloning can be employed in Caco-2 and RAW264.7 cells to better mimic the in vivo environment [66]. These strategies can enhance research methods for investigating key genes and related mechanisms in the regulation of inflammation by low-Mw fucoidan hydrolysates while offering simplicity and reliability for further research.

## 4. Conclusions

In this study, the anti-inflammatory effects and protective effects on the intestinal barrier of low-Mw hydrolysates of fucoidan were investigated in vitro using an LPS-stimulated Caco-2 and RAW264.7 co-culture model. The results showed that fucoidan, LMAF, and HMAF exhibited different immunomodulatory activity and anti-inflammatory effects. In the co-cultures, LPS significantly reduced the TEER value and induced an inflammatory response in RAW264.7 cells, leading to a significant rise in the levels of NO and cytokines TNF-α, IL-1β, IL-6, and IL-10 compared to the normal control group. Unlike fucoidan, LMAF and HMAF effectively reduced LPS-induced NO elevation. Additionally, LMAF more effectively reduced TNF-α, IL-1β, and IL-6 in the cell response triggered by LPS compared to fucoidan and HMAF. The DEGs in LPS-stimulated RAW264.7 cells were mainly enriched in the TNF signaling pathway, NF-kappa B signaling pathway, and cytokine–cytokine receptor interaction. LMAF alleviated LPS-induced inflammation by modulating the transcriptional expression of key genes such as *Tnf*, *Il6*, *Il1b*, *Serpinb2*, *Cebpb*, *Junb*, *Socs3*, and *Nfkb1* in related pathways. RT-PCR results indicated that LMAF more effectively reduced the expression levels of *Cxcl2*, *Tnf*, *Ccl2*, *Il1b*, and *Csf2*, which were significantly elevated with LPS, compared to fucoidan and HMAF. Immunofluorescence results indicated that LMAF effectively enhanced the expression levels of tight junction proteins Claudin-1, Occludin, and ZO-1 in Caco-2 cells. LMAF showed better efficacy than fucoidan and HMAF in relieving inflammation as well as enhancing intestinal barrier function. Although further carefully designed in vivo experiments are needed to explore the anti-inflammatory capacity and gut barrier-improving effects of fucoidan and its hydrolysates with different Mw, this study provides supplementary insights into the anti-inflammatory effects and immunomodulatory activity of LMAF and offers a research basis for the application of low-Mw fucoidan hydrolysates. However, the structure–activity relationship of fucoidan and its hydrolysates requires further investigation.

## Data Availability

The original contributions presented in the study are included in the article/Supplementary Materials, further inquiries can be directed to the corresponding author.

## Supplementary Materials

The following supporting information can be downloaded at: https://www.mdpi.com/article/10.3390/foods13223532/s1, Table S1: Primers sequences for RT-PCR; Table S2: The quality control of RNA sequencing of RAW264.7 cells in co-cultures.

## Abbreviations

ANOVA: one-way analysis of variance; BH, Benjamini and Hochberg; CCK8, Cell Counting Kit-8; DAPI, 4′,6-diamidino-2-phenylindole; DEGs, differentially expressed genes; DMEM, Dulbecco’s modified Eagle medium; FBS, fetal bovine serum; FC, fold change; HMAF, high-molecular-weight acidolyzed fucoidan; IL-1β, interleukin-1β, IL-6, interleukin-6, LMAF, low-molecular-weight acidolyzed fucoidan; LPS, lipopolysaccharide; MAPKs, mitogen-activated protein kinases; Mw, molecular weight; NC, normal control; NF-κB, nuclear factor-κB; NO, nitric oxide; PCA, principal component analysis; RT-PCR, real-time quantitative polymerase chain reaction; SD, standard deviation; TEER, transepithelial electrical resistance; TNF-α, tumor necrosis factor-α; TNFR1, TNF receptor 1; TNFR2, TNF receptor 2; TPM, transcripts per million reads; ZO, Zonula occluden.
