# Supplementary material for: Regulatory Effect of Fucoidan Hydrolysates on Lipopolysaccharide-Induced Inflammation and Intestinal Barrier Dysfunction in Caco-2 and RAW264.7 Cells Co-Cultures
Source: Foods. 2024 Nov 5;13(22):3532. doi: 10.3390/foods13223532 (PMC11592468; doi:10.3390/foods13223532)
Supplement: Supplementary file 1 [file foods-13-03532-s001.zip › foods-3201372-supplementary.pdf]

Regulatory effect of fucoidan hydrolysates on LPS-induced inflammation and intestinal barrier dysfunction in Caco-2 and RAW264.7 cells co-cultures

Xiaodan Fu, Xinru Huang, Huizi Tan, Xiaojun Huang, Shaoping Nie*

State Key Laboratory of Food Science and Resources, Nanchang University, China-Canada Joint Laboratory of Food Science and Technology (Nanchang), Key Laboratory of Bioactive Polysaccharides of Jiangxi Province, Nanchang 330047, Jiangxi, People's Republic of China

***Corresponding Author**

Shaoping Nie, Ph.D

Professor

State Key Laboratory of Food Science and Resources, Nanchang University, No. 235 Nanjing East Road, Nanchang 330047, Jiangxi Province, People's Republic of China

Tel.&Fax:86-791-88304452

E-mail: nie68@sina.com; spnie@ncu.edu.cn

Supplementary Table 1

Table S1 Primers sequences for RT-PCR

**Gene**	**Sequence (5’ – 3’)**	**Forward/Reverse**
IL-1β	AGTGTGGATCCCAAGCAATACCCA	F
	TGTCCTGACCACTGTTGTTTCCCA	R
TNF-α	GGTTCTGTCCCTTTCACTCACT	F
	GAGAAGAGGCTGAGACATAGGC	R
Cxcl2	CTGAACAAAGGCAAGGCTAA	F
	GCACATCAGGTACGATCCAG	R
Ccl2	AGGTGTCCCAAAGAAGCTGTA	F
	ATGTCTGGACCCATTCTTCT	R
Csf2	CCAGCTCTGAATCCAGCTTCTC	F
	TCTCTCGTTTGTCTTCCGCTGT	R
β-actin	TGGTGAAGCAGGCATCTGAG	F
	TGAAGTCGCAGGAGACAACC	R

Supplementary Table 2

Table S2 The quality control of RNA sequencing of RAW264.7 cells in co-cultures

Group	Samples	Clean reads	Error rate (%)	Q20 (%)	Q30 (%)	GC content (%)
NC	NC1	49011228	0.0130	98.15	94.30	48.95
	NC2	40597522	0.0130	98.13	94.24	49.88
	NC3	43230120	0.0129	98.17	94.40	48.59
LPS	LPS1	40492030	0.0129	98.20	94.52	48.78
	LPS2	43310504	0.0129	98.19	94.50	49.85
	LPS3	41451358	0.0130	98.15	94.35	49.41
LMAF	LMAF1	43270190	0.0130	98.17	94.39	50.07
	LMAF2	41731418	0.0129	98.20	94.46	49.56
	LMAF3	43171340	0.0129	98.21	94.51	49.44
